# Bionic Electronic Nose Based on MOS Sensors Array and Machine Learning Algorithms Used for Wine Properties Detection

**DOI:** 10.3390/s19010045

**Published:** 2018-12-22

**Authors:** Huixiang Liu, Qing Li, Bin Yan, Lei Zhang, Yu Gu

**Affiliations:** 1 School of Automation and Electrical Engineering, University of Science and Technology Beijing, Beijing 100083, China; liuhuixiang@xs.ustb.edu.cn (H.L.); liqing@ies.ustb.edu.cn (Q.L.); 2 COFCO Huaxia Greatwall Wine Co., Ltd. No. 555, Changli 066600, China; ms.yan@163.com; 3 School of Artificial Intelligence, Hebei University of Technology, Tianjin 300130, China; zhanglei@hebut.edu.cn; 4 Beijing Advanced Innovation Center for Soft Matter Science and Engineering, Beijing University of Chemical Technology, Beijing 100029, China; 5 Department of Chemistry, Institute of Inorganic and Analytical Chemisty, Goethe-University, 60438 Frankfurt, Germany

**Keywords:** portable electronic nose, wine, machine learning, support vector machine

## Abstract

In this study, a portable electronic nose (E-nose) prototype is developed using metal oxide semiconductor (MOS) sensors to detect odors of different wines. Odor detection facilitates the distinction of wines with different properties, including areas of production, vintage years, fermentation processes, and varietals. Four popular machine learning algorithms—extreme gradient boosting (XGBoost), random forest (RF), support vector machine (SVM), and backpropagation neural network (BPNN)—were used to build identification models for different classification tasks. Experimental results show that BPNN achieved the best performance, with accuracies of 94% and 92.5% in identifying production areas and varietals, respectively; and SVM achieved the best performance in identifying vintages and fermentation processes, with accuracies of 67.3% and 60.5%, respectively. Results demonstrate the effectiveness of the developed E-nose, which could be used to distinguish different wines based on their properties following selection of an optimal algorithm.

## 1. Introduction

Wine is one of the most popular drinks in the world and plays a relatively important role around the table and socially. Approximately 24.3 billion liters of wine were consumed in 2017 (International Organisation of Vine and Wine), with the United States named the world's largest consumer at 3.26 billion liters; China ranked fifth at 1.79 billion liters. Facing a vast consumer market, wine identification or classification has gained increasing popularity as a means of detecting mislabeling given the wide variability of wine sale prices depending on vintage year, fermentation processes, age, varietal, or geographical origin [1].

To assess the quality of wine in a timely manner with regard to the production process, aroma is an important indicator that cannot be ignored. Aroma is composed of hundreds of volatile chemical compounds with different concentrations that are closely related to wine attributes [2]. Typically, distinguishing wines is challenging due to the complexity and heterogeneity of its headspace [3]. However, wine classification is essential in preserving the high economic value of wine products, protecting wine quality, preventing illegal labeling, guaranteeing wine quality in the import–export market, and controlling beverage processing [4]. Although gas chromatography, mass spectrometry, and other methods are acceptable substitutes for volatile analysis of wine, they are time-consuming and labor-intensive [5].

The electronic nose (E-nose), an apparatus designed to mimic human olfactory perception, has recently become a powerful tool in the food industry [6,7,8,9,10] and other fields [11,12,13]. Regarding wine quality detection, Wei et al. [14] reported an E-nose application to distinguish between wines aged in oak barrels and others. Lozano et al. [15] developed an in-situ, on-line E-nose system for monitoring wine preservation and evolution in tanks in real time. Most relevant reports have focused on single properties of wine, whereas systematic analysis using an E-nose is lacking.

In this study, an E-nose prototype was developed to identify wines with different areas of production, vintage years, fermentation processes, and varietals. The device was mainly composed of a sensor array and a STM32F4 series-based microcontroller unit (MCU).Support vector machine (SVM) [16], random forest (RF) [17], extreme gradient boosting (XGBoost) [18], and backpropagation neural network (BPNN) algorithms were employed for pattern recognition to analyze volatile odorants in wines. The aim of this study was to classify wines with different attributes using the developed E-nose system. The remainder of this paper is organized as follows. First, we outline the development of a portable E-nose system based on a metal oxide semiconductor (MOS)-based sensor array and STM32F4 MCU; then, we present an analytical method for wine evaluation according to wine properties using the embedded E-nose system.

## 2. Materials and Methods

### 2.1. Independently Developed E-Nose Prototype

#### 2.1.1. Sensor Array

**Figure 1:**
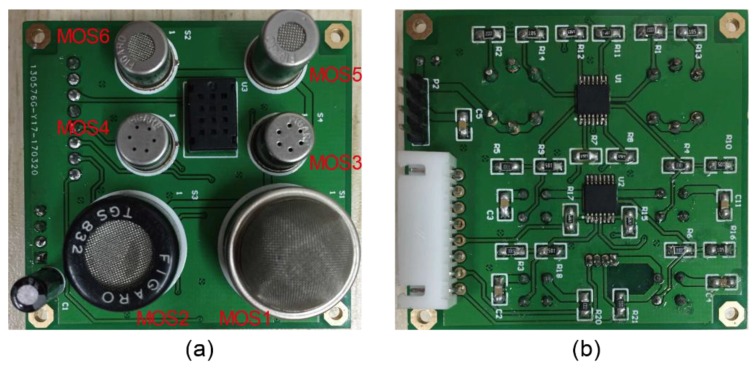
Printed circuit board (PCB) for data acquisition based on metal oxide semiconductor (MOS) sensor array. (**a**) front view; (**b**) back view.

In this work, a sensor array used in the E-nose was composed of six different metal oxide semiconductor (MOS) sensors, which were assembled in an acrylic box. The selected sensors were manufactured by Figaro Engineering Inc., Osaka, Japan. Each MOS sensor selectively adsorbed different volatile molecules during the process, resulting in conductivity changes. Therefore, a unique set of response curves from the sensor array could be obtained for each distinct object substance. The nomenclature and characteristics of the sensors are listed in Table 1. Photos of the printed circuit board (PCB), which was designed by us and produced by an original equipment manufacturer (J&C CO., LTD, Shenzhen, China), are presented in Figure 1, and it was used for data acquisition.

#### 2.1.2. Microprocessor and Peripheral Modules

In the proposed E-nose device, an STM32F407 microcontroller was used for the system and algorithm. The embedded software had two main functions: (1) acquiring sensors’ response; and (2) processing data and communicating with the computer.

Each of the sensors in the array responded to different sets of volatile organic compounds in tested substances. For subsequent computer analysis and identification, sensor responses were digitized for relevant feature extraction. A multiplexer Analog-to-Digital Converter (ADC) was thus included in the design, wherein sensor-required information was converted into a digital signal directly by the ADC according to the control signal from the MCU via the serial peripheral interface. Then, the digital signal after being processed was transmitted to the upper computer by an RS485 standard serial port for model training and testing.

**Figure 2:**
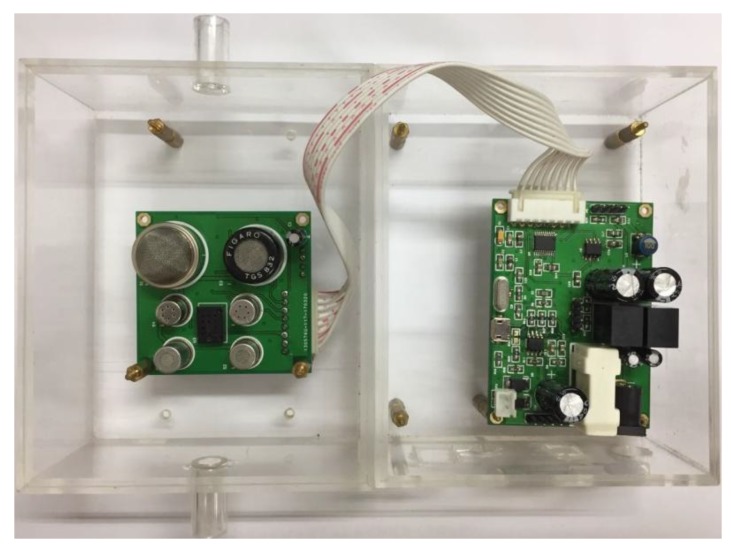
MOS-based E-nose prototype.

Considering the auxiliary heating requirements of MOS sensors, we designed a high-powered supply circuit and isolated the power supply used for auxiliary heating from the other one used for chips to enhance system stability. The other reserved interfaces or modules (e.g., debugger interface) on the PCB are not discussed in detail. The MOS-based E-nose prototype is depicted in Figure 2.

### 2.2. Wine Samples

As presented in Table 2, Table 3, Table 4 and Table 5, 14 wine samples (15 bottles per type) provided by COFCO Huaxia Greatwall Wine Co., Ltd. (in Qinhuangdao, Hebei, China) were divided into four groups to analyze their different properties (producing area, varietal, vintage, and fermentation processes). For each kind of wine, samples were taken from three different manufacturer lots. Furthermore, two of the three lots of wine were used for model training; the remaining lot was used for testing.

In the last column of Table 2, Table 3 and Table 4, “*” indicates that the processes for given samples were the same, manufacturer-announced, and details were not made public, respectively. All experiments were performed in the authors’ laboratory at a temperature of 25 ± 1 °C and a relative humidity of 50 ± 2%.

**Figure 3:**
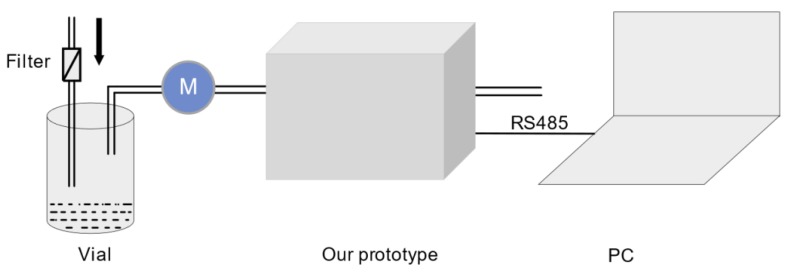
Illustration of the sample detection.

For each sample, as shown in Figure 3, 50 mL wine was put into a vial (100 ml) and was allowed to equilibrate with the air in the vial for 15 minutes. The workflow of the E-nose prototype is divided into the capturing process and the cleaning process. In the capturing process, the headspace gas of the sample is drawn into the E-nose by the flow-control unit in which they interact with the sensor array. They are adsorbed by the MOS sensors, which leads to conductivity increase and a stabilization to constant value because of the saturation of the sensor surface. During the cleaning process, air washed by carbon adsorbent is drawn into the E-nose homogeneously by the flow-control unit and analytes are removed from the sensor surface, which leads to conductivity decrease and stabilization to another constant value because of the complete removal of the analytes. Both capturing and cleaning last for 90 s.

### 2.3. Pattern Recognition Methods

#### 2.3.1. Back-Propagation Neural Network (BPNN)

BPNN is usually considered a multi-layered feedforward artificial neural network in which the backpropagation learning method is used to calculate a gradient required for calculation of the weights to be used in the network. The backpropagation process involves two stages: a feedforward stage in which exterior input information on the input nodes is propagated forward to compute output information indicators at the output unit; and a backward phase in which the connection weights are adjusted based on differences between the computed and actual indications at output units [19]. Through repeated iterations, the network’s response best matches the desired response.

#### 2.3.2. Support Vector Machines (SVMs)

SVMs are supervised learning models with associated learning algorithms that analyze data used for classification and regression analysis [16]. The SVM algorithm operates by finding the hyperplane that gives the largest margin to the training samples. Therefore, the optimal separating hyperplane maximizes the margin of the training data. For nonlinear separable classification problems, SVM applies a kernel function to transform the original space into a higher-dimensional space, and a hyperplane is constructed in the higher-dimensional space to solve problems of nonlinear separable classification in the original low-dimensional space. The four best-known kernels are linear, polynomial, radial basis function (RBF), and sigmoid [20].

#### 2.3.3. Random Forest (RF)

Random forests, or random decision forests, comprise an ensemble learning method for classification, regression, and other tasks, which operate by constructing multiple decision trees at different training times and outputting the class representing the mode of classes (classification) or mean prediction (regression) of individual trees [17]. First, for a given training set, some bootstrap samples (the amount depending on the number of classification and regression trees) were obtained by bootstrapping. Second, the RF algorithm incorporated growing classification and regression trees (CARTs). Each CART was built using random vectors. The general approach used to insert random vectors in tree formation is to choose the number of features (N_F_) in the random subset at each node, as N_F_ attributes input to be split at each node in the CART to be formed; NF can be defined using the empirical formula $N_{F}=\sqrt{M}$, where M denotes the total number of features. Finally, an RF classifier was built by growing CARTs under supervised training to determine the final classification results based on CART voting (majority rule).

#### 2.3.4. Extreme Gradient Boosting (XGBoost)

XGBoost is a scalable machine learning system for tree boosting that is a highly effective and widely used machine learning method [18]. The algorithm is based on the idea of ‘boosting’, which combines all predictions of a set of ‘weak’ learners to develop a ‘strong’ learner through additive training strategies [21]. XGBoost aims to prevent overfitting while optimizing computation resources by redefining the objective function and tree structure and optimizing the execution efficiency of the algorithm.

## 3. Results and Discussion

### 3.1. Response Curves and Features

**Figure 4:**
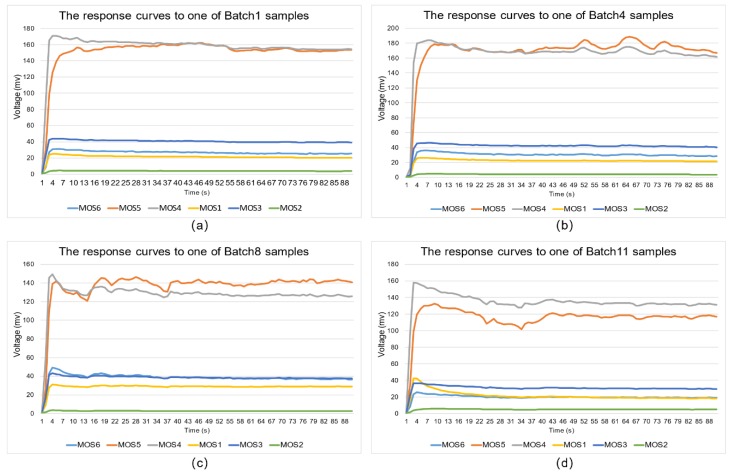
Response curves to data from different batches: (**a**) Batch 1; (**b**) Batch 4; (**c**) Batch 8; (**d**) Batch 11.

Before training and testing, sensors’ response data were analyzed and preprocessed to obtain a good feed of input features in models. Figure 4 shows the typical response signals of the sensor array to different batch samples during 90 s of measurement, respectively. Each response curve represents the voltage variation of each sensor with time when the wines’ volatiles reached the measurement chamber. The voltage value of each sensor increased rapidly and then flattened-off as the process reached steady state.

### 3.2. Principal Component Analysis (PCA) for Wine Volatiles

Principal component analysis (PCA) is widely used for feature extraction (otherwise known as dimensionality reduction) in pattern recognition. PCA is mathematically defined as an orthogonal linear transformation that transforms data to a new coordinate system such that the greatest variance by some projection of the data comes to lie on the first coordinate (the first principal component), the second greatest variance on the second coordinate, and so on [22]. In general, the first few principal components whose cumulative variance contribution exceeds 95% are considered dimensionality-reduced data and often contain nearly all information from the original data.

According to the MOS-based sensor principle, the response curve of the sensor to affinity substances quickly rises initially and then gradually flattens. Generally, several kinds of features (e.g., stable value [SV], mean-differential coefficient value, and response area value [23,24]), extracted from E-nose signals can be used in pattern recognition algorithms. In this work, we used simpler feature parameters, namely the SV. Because detection lasted for 90 s per sample and the response value of each sensor stabilized after approximately 70 s, as shown in Figure 4, the value after the 70th second of each sensor was taken as the SV. In this study, the last 10 data points (from the 81st to 90th seconds) were used as input features for model training and testing. Therefore, four datasets used for four group experiments were formed, and datasets were expressed as a 450 × 6 matrix, 600 × 6 matrix, 450 × 6 matrix, or 600 × 6 matrix, respectively.

**Figure 5:**
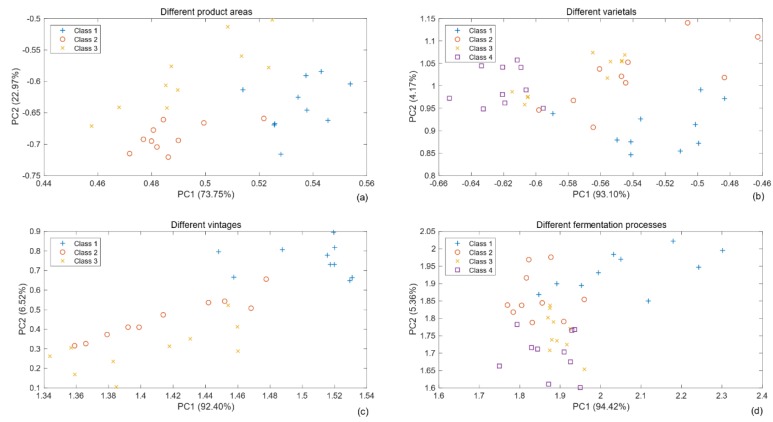
Principal component analysis (PCA) plots of different wine samples measurements (each kind of sample from the training lots were presented). (**a**) the samples with different product areas; (**b**) the samples with different varietals; (**c**) the samples with different vintages; (**d**) the samples with different fermentation processes.

PCA results of four sets of experimental data are presented in Figure 5, reducing the dimension from six variables to two principal components. The four subplots illustrate that clustering among the various classes is present, but in many cases is highly overlapping. No sets of experimental samples could be easily separated artificially in the new two-dimensional projections space based on PCA. Therefore, we concluded that the odor of wine was strong and rich; in the four sets of experiments, the sample within each group had only one different attribute, and the difference between them was minimal. In PCA, principal components with a small contribution rate were neglected, but it may reflect important differences among sample types. In particular, some useful information was lose after data dimension reduction.

**Figure 6:**
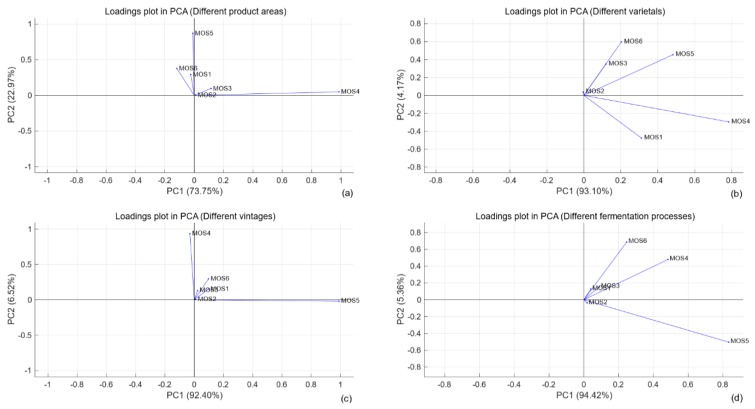
PCA loadings plots of different wine samples measurements (each kind of sample from the training lots were presented). (**a**) the samples with different product areas; (**b**) the samples with different varietals; (**c**) the samples with different vintages; (**d**) the samples with different fermentation processes.

In addition, the loadings plot of PCA is shown in Figure 6. All six variables (MOS1, …, MOS6) were represented in the subplots by a vector, respectively, and the direction and length of the vector indicate how each variable (sensor) contributes to the two principal components. In the first subplot, the first principal component had positive coefficients for the MOS2, MOS3, and MOS4, and the largest was the MOS4, which showed that MOS4 had the largest contribution to the first principal component in the process of dimension reduction. Similarly, as shown in Figure 6b–d, the sensor which had the largest contribution to the first principal component was MOS4, MOS5 and MOS5, respectively. It was worth noting that the contribution of the same sensor was different in different tasks. Actually, removing the sensors with low contribution did not improve the experimental performance in this work.

### 3.3. Comparison of Properties Classification Based on Four Methods

In this work, four methods, namely BPNN, SVM, RF, and XGBoost, were used to classify different properties of wines. The above methods were implemented via PC programming using Python language and Tensorflow (an open source software library for high-performance numerical computation). For ease of comparison, all experimental results (accuracy of the four models in different experiments, respectively) are illustrated in Table 6, where “Original”, “4-D”, and “2-D” indicate the input features used in models to be original features, 4-dimensional features reduced by PCA, and 2-dimensional features reduced by PCA, respectively.

**Figure 7:**
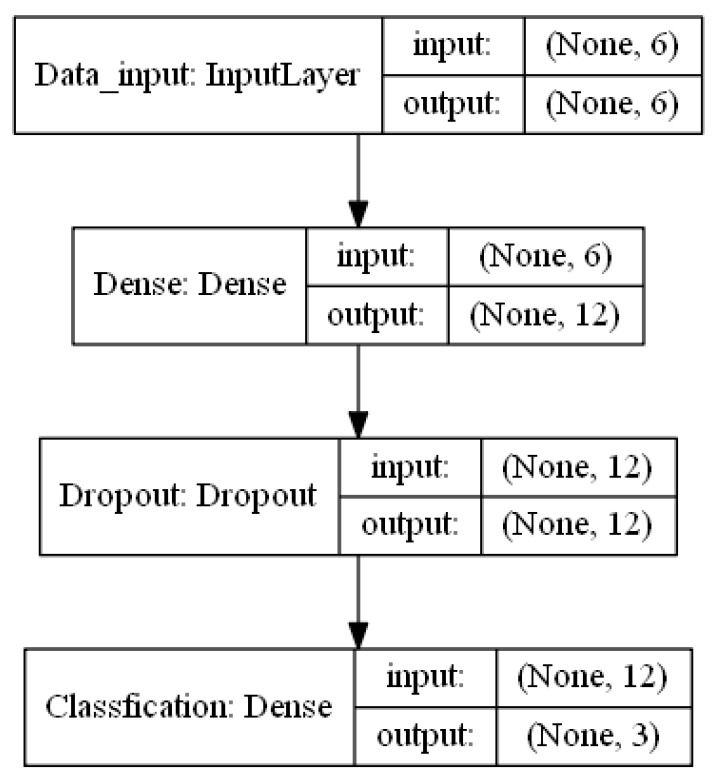
Classification model based on back-propagation neural network (BPNN).

In BPNN training, several neurons in the hidden layer were explored. During training, three-fold cross-validation was applied to evaluate generalization performance of the BPNN model with final evaluation on the testing set. Taking the first set of experiments as an example, the optimized classification model is shown in Figure 7. The number of neurons in the hidden layer was determined to be 12. During training, dropout refers to dropping out units (hidden and visible) in a neural network, involving randomly setting the fraction rate of input units to zero at each update to help to prevent overfitting.

In addition, to verify the previous guess (i.e., the contribution of small components containing important information), we compared the performance of original features and dimension-reduced features based on the model we built in each set of experiments. Experimental results are shown in Table 6. The accuracy of discrimination declined as the feature dimensions decreased. In identifying wine production areas and varietals, BPNN achieved the best performance, with accuracies of 94% and 92.5%, respectively, using original features. Results indicate that BPNN possessed strong nonlinear fitting capabilities in the two classification tasks. Note that, compared to the other methods, the training of BPNN was the most time-consuming (BPNN consumed about three to six seconds, while the others only took tens of milliseconds). Therefore, it seems futile to compare computing time when the input was so small, and we will not discuss training time hereinafter.

For the RF-based classifier model, the main parameters were the number of decision trees and number of features (N_F_) in the random subset at each node in the growing trees. During model construction, the number of decision trees was optimized first, after which the N_F_ was determined. For the number of trees, a larger amount is better but takes longer to compute. Results stop improving substantially beyond a critical number of trees, related to the N_F_ considered when splitting a node. A lower N_F_ leads to a greater reduction in variance but larger increase in bias. Only 15 decision trees were used in our experiments to build the classifier model; N_F_ was defined using the empirical formula mentioned earlier. The performance of RF is also reflected in the Table 6, revealing that the performance of RF was mediocre in all experiments.

In the SVM-based model, an RBF was chosen as the kernel function. To optimize the penalty parameter (C) and kernel parameter gamma (c) in the SVM model, a grid search method with exponentially growing sequences of C and c was applied. Then, the optimal combination of parameters was determined according to the distinguishability of the computed hyperplane. Finally, four parameter combinations ([C, c]) used for the four tasks were determined to be [10, 0.15], [10, 0.15], [20, 0.1], and [15, 0.1], respectively. According to the experimental results in the Table 6, SVM achieved the best performance in identifying wine vintage and fermentation processes, with accuracies of 67.3% and 60.5%, respectively.

XGBoost is an efficient implementation of the gradient boosting machine, a representative of ensemble learning. Model parameters were highly similar to those of the RF model, thus, the parameter selection process referenced that of the RF model. Moreover, the experimental results of the XGBoost-based method are also depicted in the Table 6. It is showed that the XGBoost model did not obtain ideal performance.

## 4. Conclusions

In this study, an E-nose prototype was developed based on MOS sensors and STM32F4 MCU. As an alternative detection method to traditional monitoring technologies, the device was employed to identify different wines. Four popular machine learning algorithms (BPNN, RF, SVM, and XGBoost) were used to build identification models for different classification tasks. Their performance was compared based on the accuracy of testing samples. The following conclusions can be drawn.

(1) PCA is unnecessary to distinguish different wines in this work, resulting in details of wine aromas being missed after the dimensions were reduced. Because the system included only six sensors, 6-dimensional features are insufficient for detailing the odor information. In particular, the accuracy of experiments with dimension-reduced samples was bad for all tasks. Overall, the distinction of vintage and fermentation processes is more difficult than producing area and varietal.

(2) Among the experimental results, BPNN and SVM performed better than RF and XGBoost; also, SVM consumed less time than the BPNN. It was found that RF and XGBoost were not good choices when dealing with low-dimensional samples, even though they performed well on many tasks. Due to the practicality of SVM, an SVM-based algorithm can be easily migrated to our developed E-nose.

(3) Results were encouraging and demonstrated that the E-nose, as a non-destructive instrument, can be used to differentiate wines when an optimal pattern recognition algorithm is selected. Although the identification accuracy of wine vintages and fermentation processes was not high enough, experiments demonstrated the effectiveness of the developed E-nose and algorithms. Insufficient samples or sensors may have affected performance; we will explore this possibility further in the next phase.

This study provides a critical outlook on the development of wine evaluation and process control, which will assist manufacturers in standardizing operation processes and reducing costs while helping to protect consumer rights.

## Figures and Tables

**Table 1:** Standard sensor array in E-nose system.

Number	Sensor	Object Substances for Sensing	Cross-Sensitive Object
MOS1	TGS826	Ammonia	Isobutane, ethanol, etc.
MOS2	TGS832	Halocarbon gas	Ethanol, R134a refrigerant, etc.
MOS3	TGS2600	Air pollutants (hydrogen, ethanol, etc.)	Isobutane, carbon monoxide, etc.
MOS4	TGS2602	Air pollutants (VOCs, ammonia, H2S, etc.)	Ammonia, hydrogen sulfide, toluene, etc.
MOS5	TGS2611	Methane	Hydrogen
MOS6	TGS2620	Alcohol, Solvent vapors	Carbon monoxide, hydrogen, etc.

**Table 2:** Details of wine samples with different producing area.

Label No.	Producing Area	Varietal	Vintage	Fermentation Processes (Yeast ID, Fermentation Container, Storage Container)
1	Huaxia	Cabernet sauvignon	2016	*
2	Renxuan	Cabernet sauvignon	2016	*
3	Zuimei	Cabernet sauvignon	2016	*

**Table 3:** Details of wine samples with different varietal.

Label No.	Producing Area	Varietal	Vintage	Fermentation Processes (Yeast ID, Fermentation Container, Storage Container)
4	Huaxia	Cabernet sauvignon	2017	*
5	Huaxia	Marselan	2017	*
6	Huaxia	Long Zibao	2017	*
7	Huaxia	Merlot	2017	*

**Table 4:** Details of wine samples with different vintage.

Label No.	Producing Area	Varietal	Vintage	Fermentation Processes (Yeast ID, Fermentation Container, Storage Container)
8	Renxuan	Marselan	2017	*
9	Renxuan	Marselan	2016	*
10	Renxuan	Marselan	2014	*

**Table 5:** Details of wine samples with different fermentation processes.

Label No.	Producing Area	Varietal	Vintage	Fermentation Processes (Yeast ID, Fermentation Container, Storage Container)
11	Huaxia	Cabernet sauvignon	2017	CC17, Stainless steel tank, Stainless steel tank
12	Huaxia	Cabernet sauvignon	2017	SC5, Stainless steel tank, Stainless steel tank
13	Huaxia	Cabernet sauvignon	2017	CC17, Stainless steel tank, Oak barrel
14	Huaxia	Cabernet sauvignon	2017	SC5, Stainless steel tank, Oak barrel

**Table 6:** Comparisons of the four methods in the classification tasks.

	Producing Area			Varietal			Vintage			Fermentation Processes		
	Original	4-D	2-D	Original	4-D	2-D	Original	4-D	2-D	Original	4-D	2-D
BPNN	**94.0**	33.3	33.3	**92.5**	50.0	46.0	52.7	32.7	30.7	52.0	38.5	50.0
RF	87.3	64.0	36.7	79.0	24.5	48.5	47.3	21.3	32.0	56.5	38.5	39.5
SVM	70.0	28.7	28.7	91.0	52.5	39.0	**67.3**	33.3	33.3	**60.5**	39.5	55.5
XGBoost	90.7	66.0	56.7	59.5	39.5	49.5	50.0	33.3	33.3	57.5	39.0	39.5

Bold values indicate the best results.
